# Spontaneous intrapartum Posterior Cul-de-sac rupture: A case report and literature review

**DOI:** 10.1016/j.amsu.2022.104572

**Published:** 2022-09-13

**Authors:** Riam Abbas, Fouad Nahhat, Maram Balouli, Heba Alsaeed, Bashar Kurdi

**Affiliations:** a University Hospital of Obstetrics and Gynecology, Damascus, Syria; b Faculty of Medicine, Damascus University, Damascus, Syria

**Keywords:** Case report, Cul-de-sac, Douglas pouch, Rupture

## Abstract

**Introduction:**

Posterior Cul-de-sac rupture is a rare delivery complication and a diagnostic challenge to every obstetrician. The associated predisposing factors include genital anomalies (such as vaginal atresia), the use of misoprostol to induce delivery, previous pelvic infection, and caesarean scar. Herein, we report the case of a posterior Cul-de-sac rupture without any disposing risk factor.

**Case presentation:**

A 27-year-old G5P4 pregnant woman at the 33rd week of gestation presented with spontaneous onset of labor, the administration of calcium channel blockers failed to stop her active labor, which progressed with a spontaneous rupture of membranes. The fetal heart rate decelerated suddenly to 40 beats per minute. Therefore, an emergency lower transverse cesarean section was performed. During the operation, a transverse 6 cm tear in the posterior vaginal wall was found. The ruptured vagina was sutured and the patient was discharged two days later in a good condition.

**Clinical discussion:**

Posterior Cul-de-sac rupture might happen without any predisposing risk factors. Also, the vague and unspecific symptoms –mainly, sudden abdominal pain-can delay the diagnosis of such an entity.

**Conclusion:**

we recommend keeping a high level of suspicion for a concealed vaginal wall rupture even in the absence of any predisposing factors, when sudden severe pain during labour cannot be otherwise explained.

## Highlights

• Posterior Cul-de-sac rupture is a rare –but serious-delivery complication.
• Posterior Cul-de-sac rupture could happen spontaneously without any predisposing risk factor.
• In most cases, the rupture manifests as sudden abdominal pain.
• The early detection and management of such cases can reduce morbidity and mortality.

## 1 Introduction

Posterior Cul-de-sac rupture is a rare delivery complication that involves the pouch of Douglas and the posterior vaginal wall. The lack of information known about this case, in addition to the vague and unspecific symptoms –mainly, sudden abdominal pain-make the diagnosis a challenge to every obstetrician and increase maternal and neonatal morbidity and mortality. Some predisposing factors mentioned in the literature include genital anomalies (such as vaginal atresia) [1], the use of misoprostol to induce delivery [2], previous pelvic infection [3], and caesarean scar [4,5]. Herein, we report the case of a woman with a spontaneous posterior Cul-de-sac rupture, without any underlying predisposing factor, to raise the awareness of such a rare entity. This work has been reported in line with the SCARE criteria [6].

## 2 Case presentation

A 27-year-old Gravid5Para4 pregnant woman presented at the emergency department of our university hospital with spontaneous onset of labour. The patient was at the 33rd week of gestation estimated on the basis of the last menstrual cycle date. Upon presentation, she had regular uterine contractions that occurred every 3–4 minutes. Ultrasound examination did not identify any abnormal findings such as oligohydramnias or fetus growth restriction. The patient did not experience neither abdominal trauma nor preeclampsia. She had earlier experienced a light, intermittent vaginal bleeding at the second trimester that was managed expectantly. The patient was not on regular visits during pregnancy, so her antenatal care was poor. Otherwise, the patient was healthy and had an unremarkable medical, drug, and family history.

Vaginal examination revealed intact membranes, 3.5 cm cervical dilation, and 65% effacement of the cervix. Transabdominal ultrasonography demonstrated a normal fetal heart rate.

**Fig. 1:**
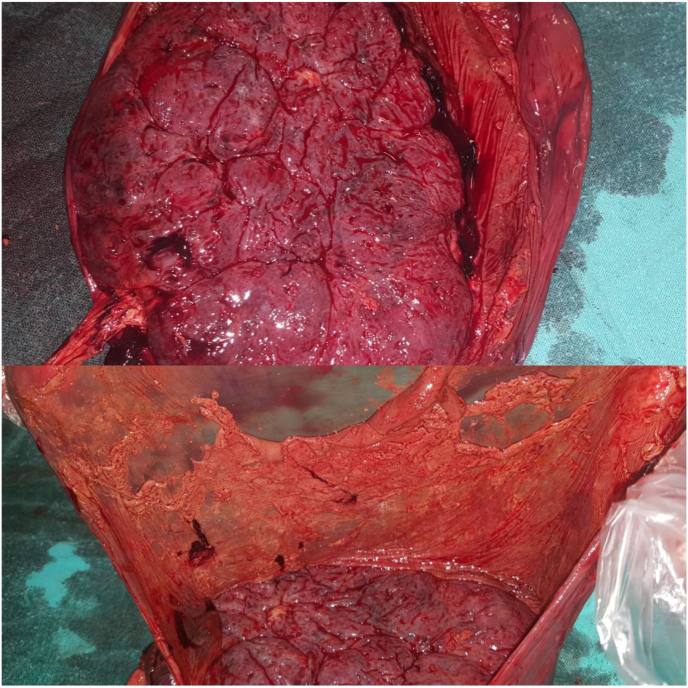
Retromembranous old looking blood clots in the placenta.

We decided to administer calcium channel blockers to stop her active labour while monitoring fetal heart rate on cardiotocography. The patient did not respond to the treatment and the preterm labour progressed with a spontaneous rupture of membranes. The amniotic fluid was bloody and contained medium-sized blood clots. Although the patient and the fetus status were reassuring, we decided to stop the administration of tocolytics and allow normal delivery to occur. However, the fetal heart rate decelerated suddenly to 40 beats per minute. Therefore, an emergency lower transverse caesarean section was performed, and a healthy baby boy was delivered successfully. The placenta was extracted and retromembranous old looking blood clots were found (Fig. 1). These findings in addition to the history of intermitted vaginal bleeding during pregnancy indicate chronic placental abruption. The uterus was closed in one layer.

**Fig. 2:**
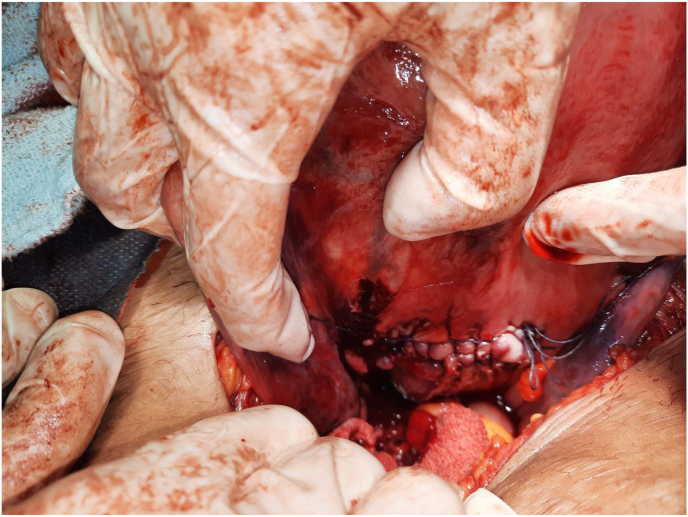
Sutured posterior vaginal wall.

The patient complained of sudden abdominal pain prior the caesarean, which was different in character from labour's pain. During the operation and upon the examination of the pouch of Douglas, a transverse 6 cm tear in the posterior vaginal wall was found. The rupture did not extend into near organs or vessels. The uterine cervix protruded through the tear toward the pouch of Douglas. We repaired the ruptured vagina with continuous Vicryl sutures in two layers, with caution to repose the cervix in its normal position (Fig. 2). A drain was left in the pouch of Douglas, and the abdomen was closed after ensuring haemostasis. She received two blood units intraoperatively. In general, the intervention was tolerable by the patient.

**Fig. 3:**
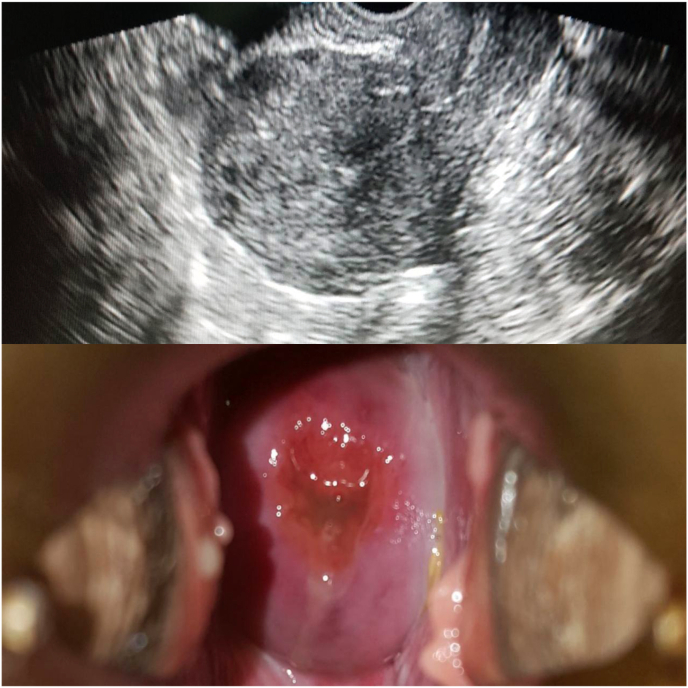
Per speculum examination and ultrasound showing normal anatomy of the cervix and vagine.

Postoperative intravenous antibiotics were given for 48 hours and continued orally for 2 weeks. The patient was hospitalized for two days and followed up for six weeks. Her recovery period was uneventful. Repeated vaginal and ultrasound examination revealed anteverted uterus and normal anatomy of the cervix and vagina (Fig. 3). Eventually, the patient was happy with the results.

## 3 Discussion and conclusion

The rupture of the posterior Cul-de-sac during labour is a rare entity with few explanation known. A review of published literature revealed 5 cases of posterior vaginal wall rupture during labour.

Young et al. reported a large laceration in the posterior vaginal wall during preterm labour at the 28th week of gestation. The patient's medical history revealed vaginal atresia, which might partially explain the resulting defect [1].

In the report of Cetinkaya et al. the patient was nulliparous at 32nd week of gestation. Her labour was induced with misoprostol for pregnancy termination due to intrauterine fetal death. The rupture was attributed to the use of misoprostol [2].

Bertaud and Ratto correlated the rupture of the posterior Cul-de-sac with a history of previous pelvic infection, which changed the elastic properties of the birth canal [3]. Since our patient is 27-year old G5P4 pregnant woman, then a previous pelvic infection cannot be excluded. But, the patient did not experience neither a lower abdominal pain nor purulent cervical and/or vaginal discharge previously, so it is unlikely that she had previous pelvic infection.

In the other cases, the spontaneous rupture happened in a scarred uterus due to previous caesarean sections [4,5]. It was thought that the fibrotic nature of the uterine scar resulted in a higher mechanical tension on the posterior wall, which might explain the rupture [7].

There are some anatomical causes of the poterior cul-de-sac rupture that must be mentioned such as the marked obliquity of uterine axis, uterine deflexion, and weakening of the vaginal wall. None of the above was found in our patient.

While the previously reported cases had potential predisposing factors (such as vaginal atresia and a previous pelvic infection), the rupture in our patient occurred spontaneously in absence of any known predisposing factor. The only factor that merits consideration in our case is the chronic abruption of the placenta. However, we believe that it is very unlikely to contribute to the rupture of the posterior Cul-de-sac. Also a similarity between our case and the first two cases is the preterm labour but we did not find it to have a proven correlation to the rupture. It is noteworthy that the patient did not have congenital Müllerian anomalies, and we didn't use neither oxytocin nor prostaglandin for labour augmentation. Therefore, it is not clear why the tear occurred in this case.

On the other hand, the common symptom reported in the literature and in our case is sudden abdominal pain, which differs from the pain caused by active uterine contractions. The absence of specific symptoms can delay the diagnosis.

Thus, we recommend keeping a high level of suspicion for a concealed vaginal wall rupture even in the absence of any predisposing factors, when sudden severe pain during labour cannot be otherwise explained.

## Ethical approval

No ethical approval was needed.

## Funding sources

There were no sources of funding.

## Authors contribution

Riam Abbas: Participated in surgical procedure and wrote the case presentation.

Fouad Nahhat: Wrote the introduction and discussion.

Maram Balouli: Reviewed the literature and wrote the abstract.

Heba Alsaeed: Reviewed the literature and designed the figures.

Bashar Kurdi: Led the surgical procedure and supervised the writing of the manuscript.

## Patient's consent

Written informed consent was obtained from the patient for publication of this case report and accompanying images. A copy of the written consent is available for review by the Editor-in-Chief of this journal on request.

## Guarantor

Mr. Fouad Nahhat.

## Data availability

All data are included in this article. Further enquiries can be directed to the corresponding author.

## Declaration of competing interest

All authors declared that they have no conflicts of interest.
